# Bioinformatics analysis combined with experiments predicts CENPK as a potential prognostic factor for lung adenocarcinoma

**DOI:** 10.1186/s12935-021-01760-y

**Published:** 2021-01-21

**Authors:** Jiayu Ma, Xiaochuan Chen, Mingqiang Lin, Zhiping Wang, Yahua Wu, Jiancheng Li

**Affiliations:** 1 Department of Radiation Oncology, Fujian Cancer Hospital & Fujian Medical University Cancer Hospital, No.420, Fuma Road, Fuzhou, 350014 China; 2 Fujian Medical University, Fuzhou, Fujian China

**Keywords:** Lung adenocarcinoma, CENPK, Novel biomarkers, Prognosis

## Abstract

**Background:**

Lung cancer is the most common malignant tumor. Identification of novel diagnostic and prognostic biomarkers for lung cancer is a key research imperative. The role of centromere protein K (CENPK) in cancer is an emerging research hotspot. However, the role of CENPK in the progression of lung adenocarcinoma (LAC) is not well characterized.

**Methods:**

In this study, we identified CENPK as a potential new gene for lung cancer based on bioinformatics analysis. In addition, in vitro experiments were performed to verify the function of this gene. We investigated the expression of CENPK in LAC by analyses of datasets from The Cancer Genome Atlas (TCGA) and Gene Expression Omnibus (GEO) databases. Differential expression analyses, gene ontology (GO) enrichment, Kyoto encyclopedia of genes and genomes (KEGG) analysis, and gene set enrichment analysis (GSEA) were conducted to evaluate the diagnostic and prognostic relevance of CENPK. Then, for evaluating the biological behavior and role of CENPK in lung cancer cells, we did a series of vitro experiments, such as immunohistochemistry analysis, Western blot analysis, CCK8 assay, transwell assay, flow cytometry, and wound healing assay.

**Results:**

We demonstrated overexpression of CENPK in LAC; in addition, increased expression of CENPK was associated with clinical progression. Moreover, CENPK was found to be an independent risk factor in patients with LAC. Furthermore, we observed activation of CENPK-related signaling pathways in patients with LAC.

**Conclusions:**

Our findings indicate a potential role of CENPK in promoting tumor proliferation, invasion, and metastasis. It may serve as a novel diagnostic and prognostic biomarker in patients with LAC.

## Introduction

Globally, an estimated 18.1 million new cancer cases and 9.6 million cancer-related deaths occurred in 2018. Lung cancer is the most frequently diagnosed cancer and the main cause of cancer deaths in both men and women [1]. Despite several advances in staging, diagnostic procedures, and treatment options, the general outlook for most patients with lung cancer has not changed much. Over the past decade, there has been only a marginal increase in the 5-year overall survival rate of patients with lung cancer (from 15.7 to 17.4%) [2]. There are two main subtypes of lung cancer, non-small cell lung cancer (NSCLC) accounts for approximately 80–85% of all lung cancer cases while small cell lung cancer (SCLC) accounts for the remaining cases [3, 4]. There are three subtypes of NSCLC: squamous-cell carcinoma, adenocarcinoma, and large-cell carcinoma [5, 6]. The most common type of NSCLC is adenocarcinoma which accounts for approximately 40% of all lung cancers. Adenocarcinoma originates from small alveolar epithelial cells of the type II airways [7]. The currently available treatment modalities for lung cancer include surgery, chemotherapy, radiation therapy, and molecular targeted therapy. The advances in genetic testing have helped identify specific mutations to better target therapy [8]. Despite the use of biomarkers and immunotherapy (PD-1 and PD-L1 therapies), the average overall survival rates continue to be poor [9].

Human centromere protein K (CENPK) (also known as *AF5alpha, FKSG14, P33, ICEN37*, and Sauer) is a protein encoded by the *CENPK* gene (located on chromosome 5q12.3). It consists of 269 amino acids and has a molecular weight of 31 kD [10, 11]. Kinetochore is a protein structure on chromatids, comprising of at least 80 different proteins, which plays an important role in the separation of chromosomes in all eukaryotes [12]. Gene annotation enrichment analyses have shown the involvement of CENPK subgroups in mitosis in the cell cycle. An increasing body of evidence has implicated dysregulation or dysfunction of CENPK in cancer progression [13]. Overexpression of CENPK has been demonstrated in various human malignancies including hepatocellular carcinoma [14, 15], ovarian cancer [16], and breast cancer [17]. Therefore, we speculate that CENPK is a potential new oncogene. However, the relationship between the CENPK gene and the development of lung adenocarcinoma (LAC) is not well characterized in the contemporary literature.

In the present study, we identified overexpression of CENPK in LAC; in addition, high CENPK expression showed an association with tumor progression. Besides, we determined that CENPK may be a useful diagnostic and prognostic biomarker. Moreover, transcriptional expression of CENPK was found to be independent risk factor for survival of patients with LAC. Additionally, cell function experiments verified the biological behavior of CENPK in lung cancer. Our findings shed light on the crucial role of CENPK in LAC as well as provide a potential diagnostic and prognostic biomarker.

## Methods

### Data acquisition and processing

We downloaded gene expression data and the corresponding clinical information from the Cancer Genome Atlas (TCGA) data portal (https://tcga-data.nci.nih.gov/tcga/) available as of 1 October 2019. Data pertaining to a total of 535 tumor samples and 59 peritumoral normal tissues were included. This project was conducted according to the guidelines provided by TCGA. The statistics and details are provided in Additional file 1: Table S1 and Additional file 2: Table S2.

The Gene Expression Omnibus (GEO) database (www.ncbi.nlm.nih.gov/geo) was used for the validation. The GSE72094 dataset which includes data pertaining to 442 LAC samples was downloaded to investigate the relation between CENPK expression and prognosis. The statistics and details are provided in Additional file 1: Table S3 and Additional file 3: Table S4.

### Gene and functional set enrichment analysis

Gene set enrichment analysis (GSEA) was used to detect whether a priori defined set of genes showed significant differential expression between the high and low CENPK expression groups in the enrichment of MSigDB Collection [18, 19]. In our study, GSEA first generated an ordered list of all genes according to their correlation with CENPK expression. GSEA was carried out to access the significance of the observed difference between the survival outcomes in the high- and low-CENPK groups. CENPK expression level was used as a phenotype label. The nominal p value and normalized enrichment score (NES) were evaluated to sort the pathways enriched in each phenotype. Gene set permutations were performed 1000 times for each analysis. Results are presented using the grid and gridExtra R package [20].

### Functional enrichment analysis

gene ontology (GO) and Kyoto encyclopedia of genes and genomes (KEGG) analyses were performed to determine the potential biological functions of CENPK. Genes whose expression correlated with that of CENPK (adjusted p < 0.05 and |log FC|≥ 1) were considered to be risk score-associated genes and were subjected to GO and KEGG analyses to identify the potential biological functions and pathways. In R software, the “ClusterProfiler” package was utilized to analyze GO and KEGG pathways [20]. GO and KEGG enrichment analysis was according to the threshold of p < 0.05 and q < 1.

### Immunohistochemistry analysis

The slides were analyzed by immunohistochemistry with anti-human CENPK (catalog no. 26208-1-AP), followed by HRP secondary antibody. Microscopic images were obtained for analysis. Histochemistry score (H-score) was used to quantify the expression of CENPK using the following formula: H-score = (weak intensity% × 1) + (moderate intensity% × 2) + (strong intensity% × 3). The details and statistics of patient information in the immunohistochemistry slides are provided in Additional file 4: Table S5 and Additional file 1: Table S6.

### Cell lines and cell culture

Normal lung epithelium cell line human bronchial epithelial (HBE), NSCLC cell line A427 and LAC cell lines A549 and H1299 were obtained from the Genechem company (Shanghai, China). All cell lines were grown in RPMI-1640 supplemented with 10% FBS and 0.01 mg/mL recombinant human insulin in a humidified incubator with 5% CO_2_ at 37 °C.

### Western blot analysis

Cells were washed with PBS and lysed using RIPA extraction reagent; the protein concentration was determined using the Bicinchoninic Acid (BCA) protein assay kit. Samples were subjected to SDS-PAGE and electrophoretically transferred to PVDF membranes. Subsequently, the membranes were blocked with 2% BSA in TBST, and incubated overnight with primary antibodies at 4 °C followed by incubation with secondary antibody at room temperature for 1 h. Samples were washed with TBST prior to their visualization by chemiluminescence.

### Cell transfection

We chose cells in the logarithmic growth phase. siRNAs were used to selectively knockdown the CENPK gene. The CENPK siRNAs (si-GAP, si-341, si-550, si-944), and the siRNA negative control (NC) were obtained from the Genepharma Biotechnology (Xiamen, China). According to the manufacturer’s instructions, we chose the best transfection efficiency which was designed to selectively knockdown the expression of CENPK. Cells were allowed to achieve a 40–50% confluence and seeded overnight to adhere to the wall prior to transfection. siRNAs were mixed with Lipofectamine RNAiMAX (Invitrogen, USA) in opti-MEM. The efficiency of siRNA knockdown was verified by Western blot analysis.

### CCK8 assay

Cell Counting Kit-8 (CCK8) assay (Beyotime, China) was used to measure cell viability. After transfection, cells were placed in 96-well plates at a density of 500 cells per well; the absorbance values were detected 0 to 4 days after transfection. 10 μL of CCK8 solution was added daily to each well filled with 100 µL RPMI 1640 medium in the 96-well plates and incubated for another 2 h. Then, a microplate reader (Bio-Rad, USA) was used to measure the absorbance at 570 nm.

### Transwell assay

The cells were digested and configured to a density of 1 × 10^6^/mL. In the upper chamber of the Transwell (Millicell, Merck company, Germany) 100 μL of the adjusted concentration of cell suspension was added, while the lower chamber was filled with 1640 medium including 20% FBS; subsequently, the plate was placed in the incubator. The migration and invasive capability of cells was assessed based on the penetration of the membrane and gel membrane of the matrix, respectively. After 1 day, the residual cells in the upper chamber were wiped with cotton swabs and dried. The sample was fixed with 4% paraformaldehyde, washed with PBS, then mixed with crystal violet for a quarter, then washed by PBS. After the filter membrane had dried, photographs were obtained using an upright microscope.

### Flow cytometry

For cell cycle analysis, 1 × 10^6^/mL cells were counted and washed with PBS, followed by addition of 70% chilled ethanol and overnight placement at 4 °C; subsequently, the cells were washed again, centrifuged after re-suspension, and cycle detection reagent without RNA enzyme (550825, BD, USA) was added for 30 min. The data were analyzed using ModFit LT 2.0. Cell apoptosis was assessed using the Apoptosis Detection Kit (559763, BD, USA) after the cell count had reached 1 × 10^5^. The data were analyzed with cflow 1.4. Cell cycle and cell apoptosis were measured using flow cytometer (BD Accuri C6, Fisherbrand Company, USA).

### Wound healing assay

After digestion, 1 × 10^5^ cells were counted. After 48 h of transfection, when the cells had achieved confluence, the cell monolayer was scraped with the tip of a yellow pipette (200 µL) and photographs obtained with an inverted microscope. The plate was placed in the incubator for 24 h; subsequently, photographs were obtained.

### Statistical analysis

The expression of CENPK was analyzed using the Wilcoxon signed-rank test. The association between clinical-pathological features and CENPK expression was evaluated using the Wilcoxon signed-rank test or the Kruskal–Wallis test. Overall survival (OS) was compared between the high and low CENPK expression groups through Kaplan–Meier analysis using the Survival and Survminer package in R [21, 22]. Univariate Cox analysis was used to identify the potential prognostic factors. Multivariate Cox analysis was performed to assess whether make sure CENPK was an independent risk factor for OS of patients with LAC. Comparisons between two groups were analyzed by t test. All data analyses were performed using the R software (version 3.6.3), GraphPad 7.0, ImageJ 1.48 and Adobe Illustrator CS6.

## Results

### Over-expression of CENPK in lung adenocarcinoma

Data from TCGA revealed overexpression of CENPK in LAC samples (n = 535) compared with the non-tumor samples (n = 59). We downloaded data pertaining to age, sex, smoking history, histologic grade, clinical stage, TNM classification, and recurrence of LAC; finally, we selected 399 LAC samples with complete clinical information and 398 LAC samples from the GEO database (GSE72094) for validation.

**Fig. 1:**
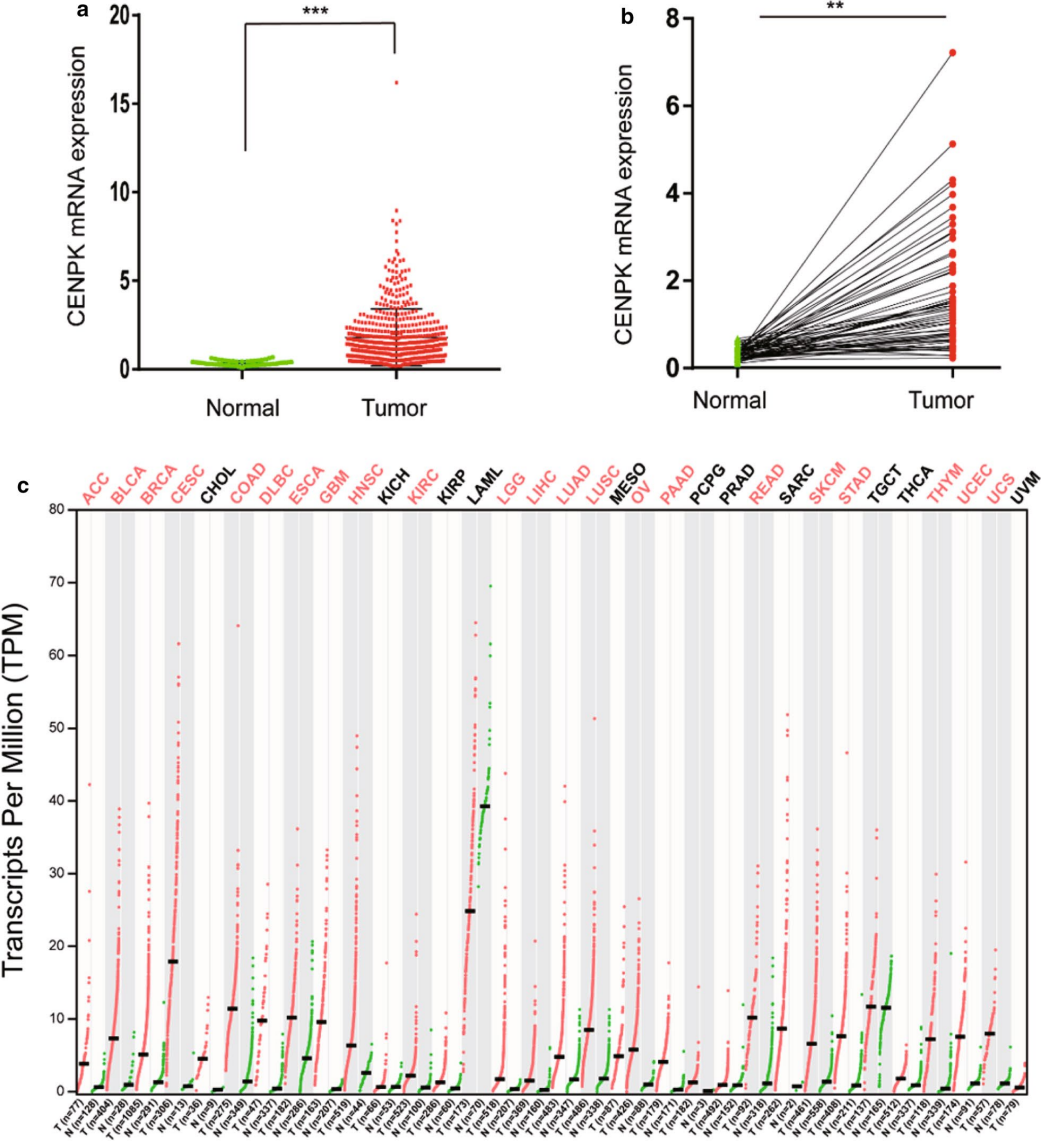
RNA-seq data of tumor tissues and peritumoral normal tissues. **a**, **b** Data from TCGA showed significantly higher CENPK mRNA expression in lung adenocarcinoma (N = 535) than that in normal tissues (N = 59). **c** Different expression in various cancers by RNASeq were obtained from the TCGA portal. Red represents a difference; black indicates no difference. *P < 0.05, **P < 0.01, ***P < 0.001

We compared the mRNA expression levels of CENPK between LAC samples and peritumoral normal tissues. We observed overexpression of CENPK in lung adenocarcinoma Fig. 1a. CENPK mRNA expression in tumor tissues was significantly increased compared to that in the paired peritumoral normal tissues Fig. 1b. Currently, the relationship between CENPK and oncogenesis is not well characterized. To determine the expression of CENPK in other cancers, we performed a comprehensive analysis of 33 types of tumors from TCGA. Among these, 22 types of tumors showed overexpression of CENPK Fig. 1c.

### Over-expression of CENPK is associated with advanced lung adenocarcinoma

**Fig. 2:**
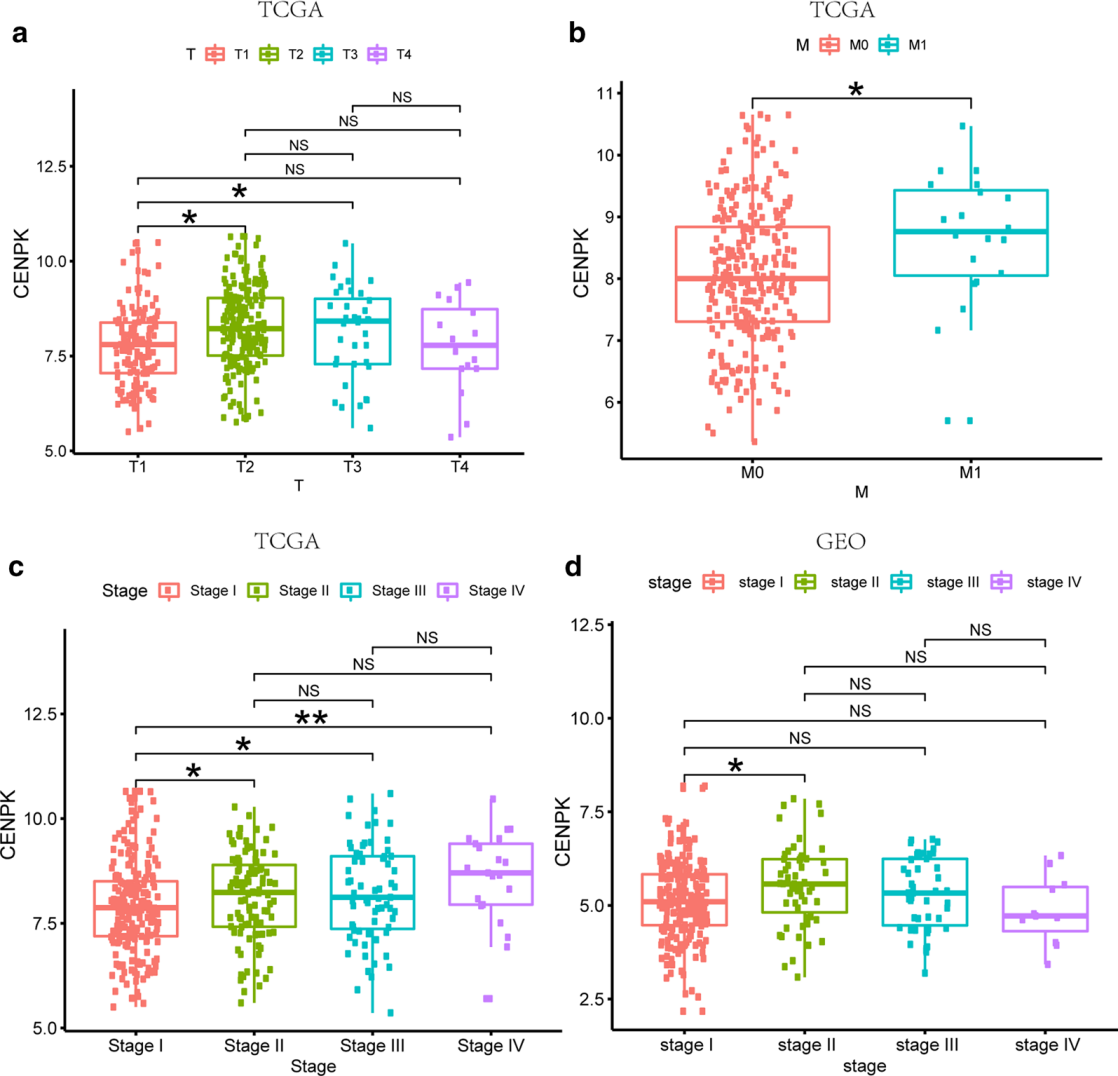
CENPK expression in lung adenocarcinoma. **a** T stage in TCGA database (T1 = 139, T2 = 208, T3 = 36, T4 = 16); **b** metastasis classification (metastasis negative n = 272, and metastasis positive n = 18); **c** clinical stage, including stage I (n = 139), stage II (n = 208), stage III (n = 36), and stage IV (n = 16); **d** clinical stage in GEO database, including stage I (n = 239), stage II (n = 62), stage III (n = 61), and stage IV (n = 16)

We analyzed the association between mRNA expression of CENPK and clinical-pathological parameters of patients with LAC, including cancer stage, T stage, lymphatic metastasis, distant metastasis, sex, and age. mRNA expressions of CENPK showed a remarkable association with T stage Fig. 2a, and distant metastasis Fig. 2b. Patients with more advanced disease stage tended to exhibit higher mRNA expression of CENPK. Moreover, subgroup analysis revealed a relationship of CENPK expression with cancer stage Fig. 2c. Of note, there was no apparent difference in CENPK expression in LAC with lymphatic metastasis, this phenomenon may be attributable to the distribution of cases in the TCGA database, more than 60% of patients were in N0 stage, no more than 20% and 15% of patients were in N1 and N2 stages, and only two patients were in N3 stage. This extremely unbalanced number of cases makes it impossible to draw more objective conclusions.

In the GSE72094 dataset from the GEO database, we assessed the association between CENPK and clinical characteristics, especially clinical stage. Patients with more advanced cancer stage tended to express higher mRNA and protein levels of CENPK Fig. 2d. These results suggested that compared with low CENPK expression, patients with LAC who have high CENPK expression are likely to have poor prognosis.

### Over-expression of CENPK was associated with poor prognosis of LAC patients

**Fig. 3:**
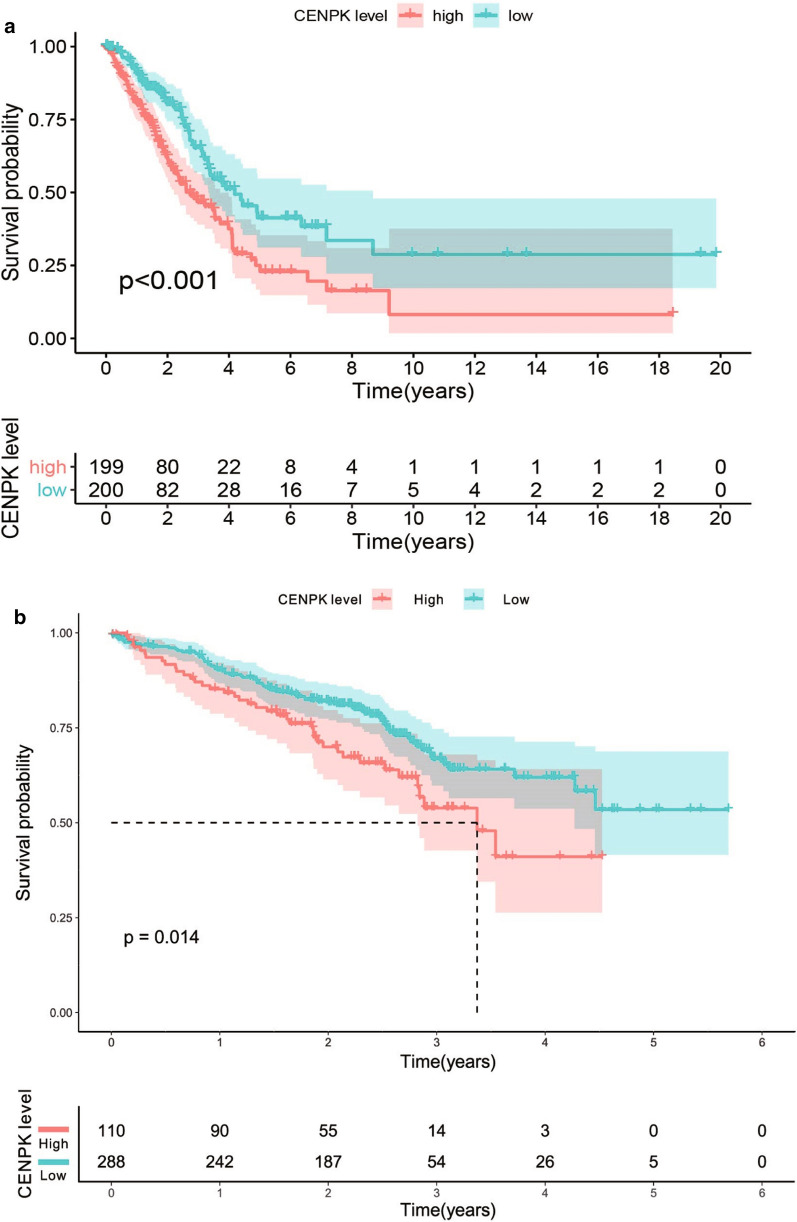
Kaplan–Meier curves for survival probability in lung adenocarcinoma. High CENPK expression was related to poor survival probability. **a** Data from TCGA database (P < 0.001). **b** Data from GEO (P = 0.014, log-rank test; cut-off value: 2.78)

We assessed the prognostic value of CENPK mRNA expression in patients with lung adenocarcinoma using Kaplan–Meier survival analysis. Higher CENPK mRNA expression showed a remarkable association with poor survival in LAC patients Fig. 3a. We verified in the GEO database and drew the same conclusion Fig. 3b. In our study, the cut-off level to determine high and low expression of CENPK was based on the Youden Index (overexpression group ≥ 2.78, low expression group < 2.78). Twenty-eight percent of patients had low expression of CENPK while 72% had high expression of CENPK. These results indicated that mRNA expression of CENPK was closely related to the prognosis of patients with LAC. Thus, CENPK is a potential biomarker for predicting the survival of these patients.

### High CENPK expression was an independent risk factor for survival of LAC patients

**Fig. 4:**
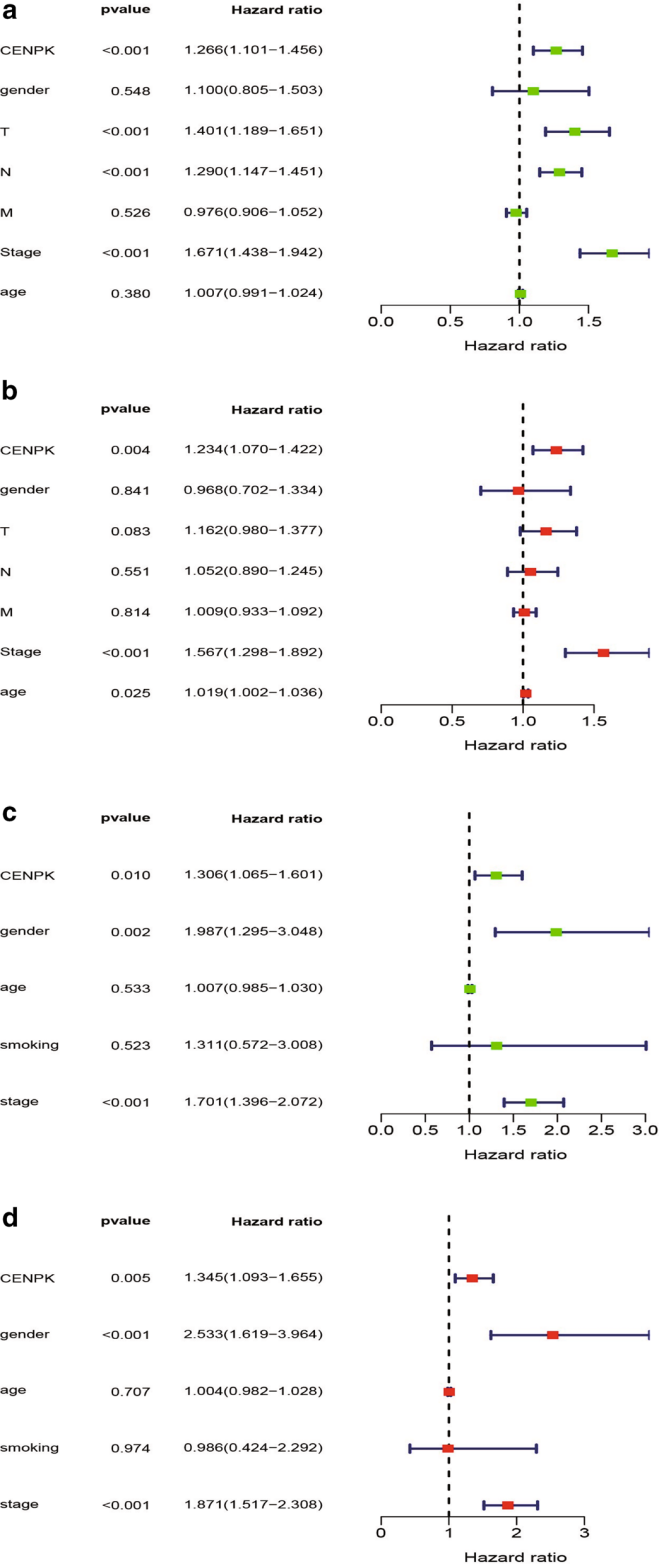
Forest map showing high CENPK expression as an independent risk factor. Results of univariate and multivariate analysis showing the independent predictive ability of CENPK for OS. **a** Univariate analysis of TCGA database. **b** Multivariate analysis of TCGA database. **c** Univariate analysis of GEO database. **d** Multivariate analysis of GEO database. HR > 1 can be used as an independent risk factor

We performed univariate and multivariate Cox analysis to evaluate the impact of CENPK expression on the survival of patients with LAC. On univariate analysis, CENPK-high expression showed a significant correlation with poor survival probability [hazard ratio (HR): 1.266, 95% confidence interval (CI) 1.101–1.456; p < 0.001]; in addition, the T stage, N stage, and clinical stage were also included Fig. 4a. On multivariate Cox analysis, high CENPK expression showed a correlation with poor survival probability (HR: 1.234, 95% CI 1.070–1.422, p = 0.004) Fig. 4b. Other variables related to poor survival included the clinical stage. The results from GEO also supported this conclusion. On univariate analysis, high expression of CENPK was associated with poor survival probability (HR: 1.306, 95% CI 1.065–1.601, p = 0.010) same as sex and stage Fig. 4c, multivariate analysis revealed the same result (high expression of CENPK, HR: 1.345, 95% CI 1.093–1.655, p = 0.005) Fig. 4d.

### GSEA to identify CENPK-related signaling pathways

**Fig. 5:**
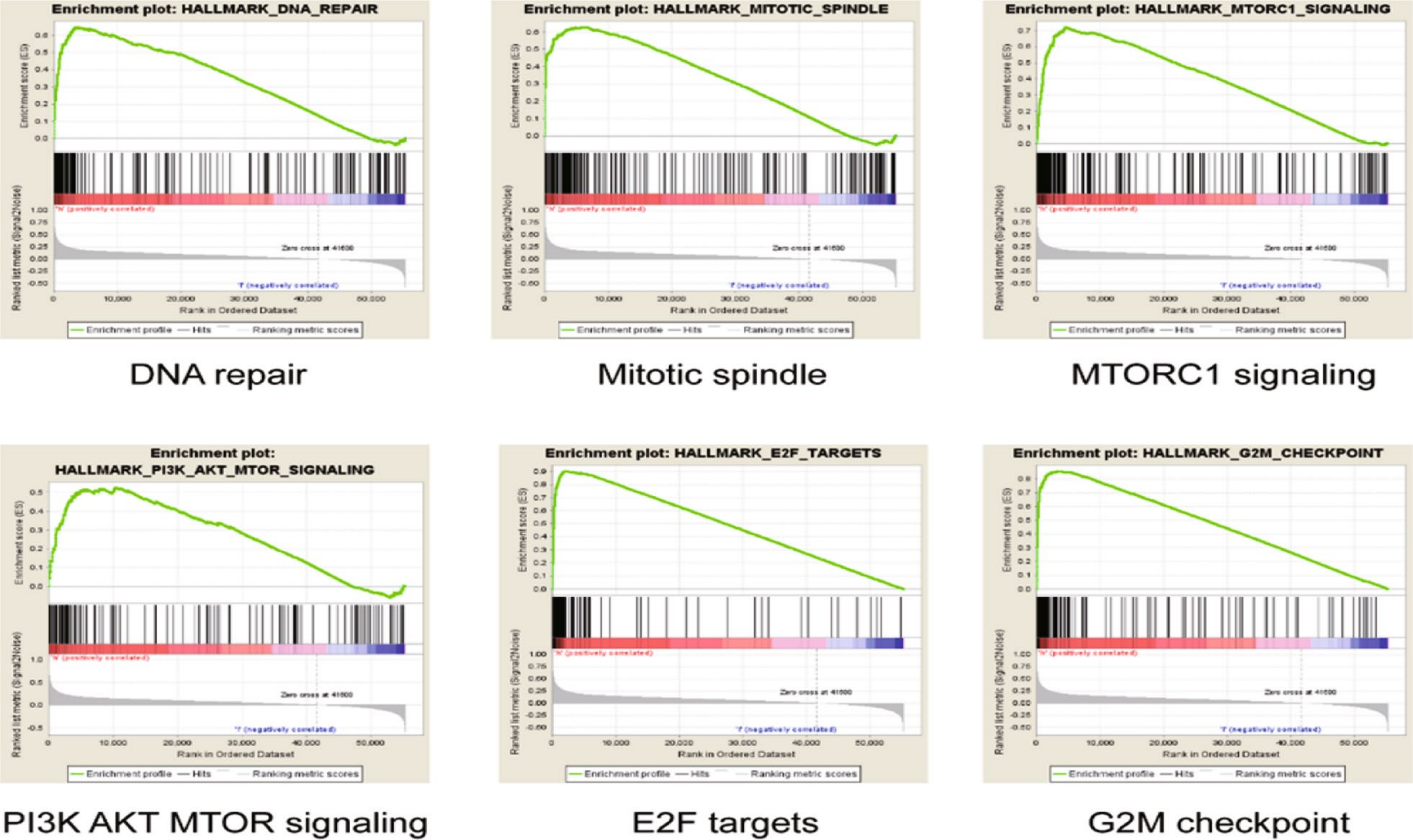
Enrichment plots from GSEA. GSEA results showing differential enrichment of genes in lung adenocarcinoma cases with high CENPK expression, including DNA repair, mitotic spindles, PI3K AKT MOTR signaling, E2F targets, G2M checkpoint

To identify the signaling pathways which are activated in LAC, we performed GSEA to compare the high and low CENPK expression groups. Gene sets enriched in high CENPK expression groups were related to DNA repair, mitotic spindles, PI3K AKT MOTR signaling, E2F targets, G2M checkpoint; these pathways mainly stimulate tumor proliferation, invasion, and metastasis Fig. 5. It is believed that CENPK may modulate the tumor-related signaling pathways to influence cell proliferation and promote the occurrence and development of LAC.

### KEGG and GO analysis to identify CENPK-related signaling pathways

**Fig. 6:**
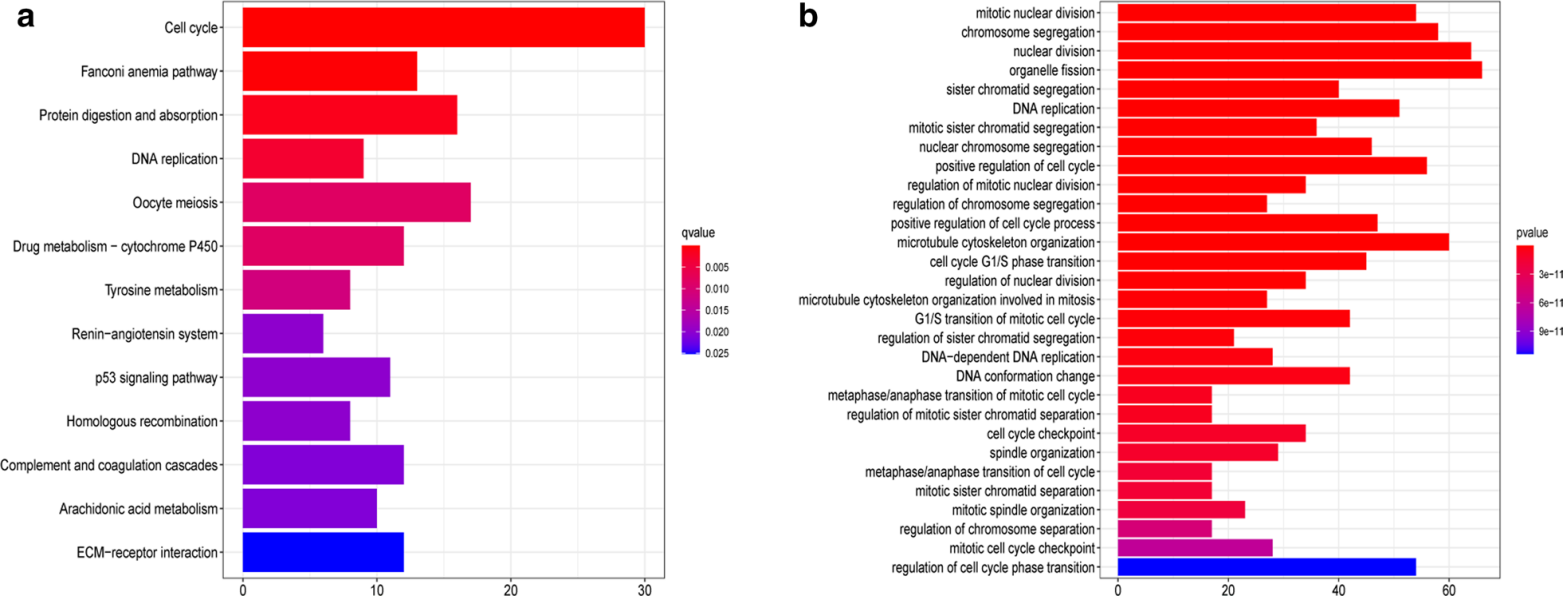
KEGG and GO analysis to identify CENPK-related signaling pathways. **a** KEGG signaling pathway enrichment analysis; **b** Gene ontology pathway enrichment analysis. In the histogram, the abscissa represents the proportion of genes, and the ordinate represent the enriched pathway. For example, the length of the pathway band on the right side of the abscissa represents enrichment of a greater number of genes. At the same time, compared to the purple band, red indicates that the differential enrichment is more significant

We conducted GO and KEGG enrichment analyses to elucidate the functions and signaling pathways of the genes co-expressed with CENPK. GO and KEGG terms were visualized using the ggplot2 R software package. High CENPK expression groups were related to the cell cycle, oocyte meiosis, protein digestion and absorption, DNA replication, and other pathways in KEGG Fig. 6a. In line with KEGG results, GO functional analysis also revealed that the co-expressed genes were associated with nuclear division, microtubule cytoskeleton organization, chromosome segregation, and mitotic nuclear division Fig. 6b, all of which mainly stimulate tumor proliferation, invasion, and metastasis. These pathways related to the cell cycle, DNA replication, and mitotic spindle, were also consistent with GSEA.

### Immunohistochemistry to verify CENPK expression

**Fig. 7:**
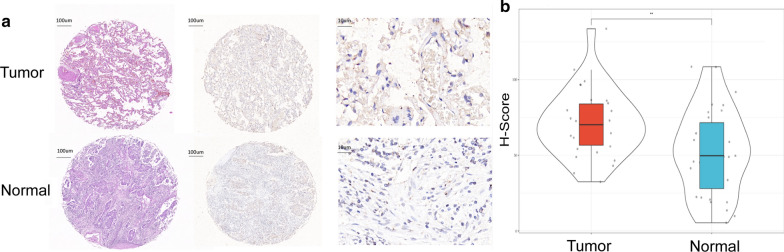
CENPK staining in lung adenocarcinoma and normal tissues. **a** Lung adenocarcinoma microarray and normal tissue immunohistochemistry contrast HE staining (× 10), immunohistochemistry (× 10, × 100). **b** Statistical graphics quantifying the expression of CENPK by H-score. H-score = (weak intensity% × 1) + (moderate intensity% × 2) + (strong intensity% × 3)

Tissue microarrays containing lung cancer tissues and para-cancerous tissues and the associated clinical information was obtained from service company (Wuhan, China); data pertaining to LAC was extracted. The details are provided in Additional file 1: Table S3. Lung adenocarcinoma microarray and peritumoral normal tissue immunohistochemistry contrast HE staining (× 10), and immunohistochemistry (× 10, × 100) are shown in Fig. 7a, H-score statistics are shown in Fig. 7b.

### Verification of the expression of CENPK and related pathways in vitro

**Fig. 8:**
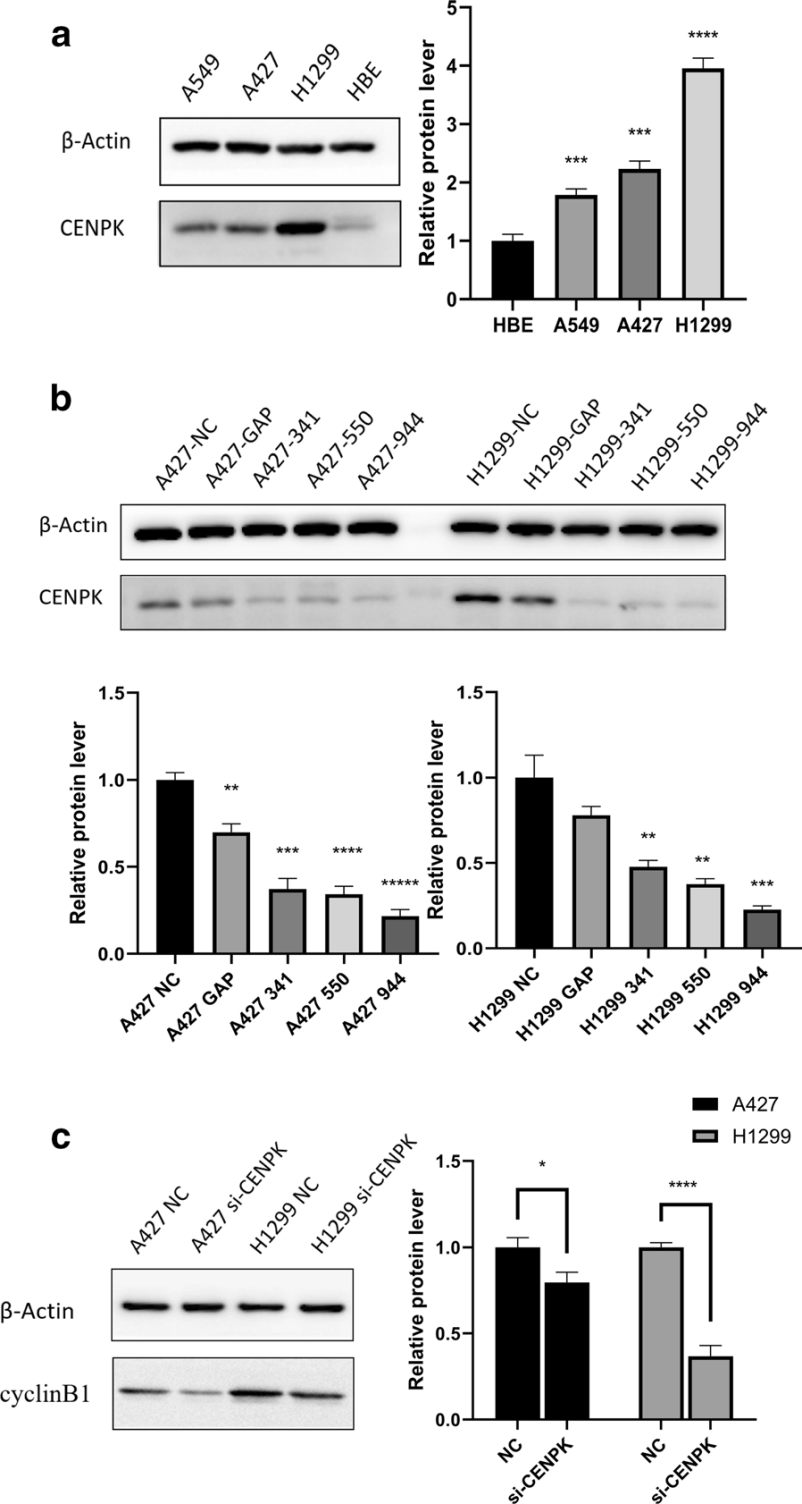
In vitro verification of the expression of CENPK and related pathways. **a** Western blotting analysis of CENPK expression in HBE cells and three NSCLC cell lines. **b** siRNAs were used to selectively knockdown CENPK genes in A427 and H1299 cells. **c** Protein expression changes of cyclinB1

To choose the proper cell model for follow-up research, we compared the protein expression of CENPK in different cells, including HBE cell, adenocarcinoma cell lines (A549 and H1299) and NSCLC cell line (A427). HBE exhibited the lowest expression level of CENPK, while H1299 and A427 showed higher expression; therefore, the latter two cell lines were selected for the subsequent experiments Fig. 8a. We constructed A427 and H1299 cells by endogenously knocking down CENPK by transfection of specific siRNAs, named as A427 si-CENPK and H1299 si-CENPK Fig. 8b. In the Western-blot assay, we validated the proteins related to cell cycle pathways, we found that knockdown of CENPK was significantly related to cyclinB1 (proteintech, catalog no. 26208-1-AP) Fig. 8c, which is the critical protein in the G2 phase. This was most obvious in KEGG analysis, and other cycle related protein are provided in Additional file 5: Fig S1.

### Knockdown of CENPK inhibited cell proliferation

**Fig. 9:**
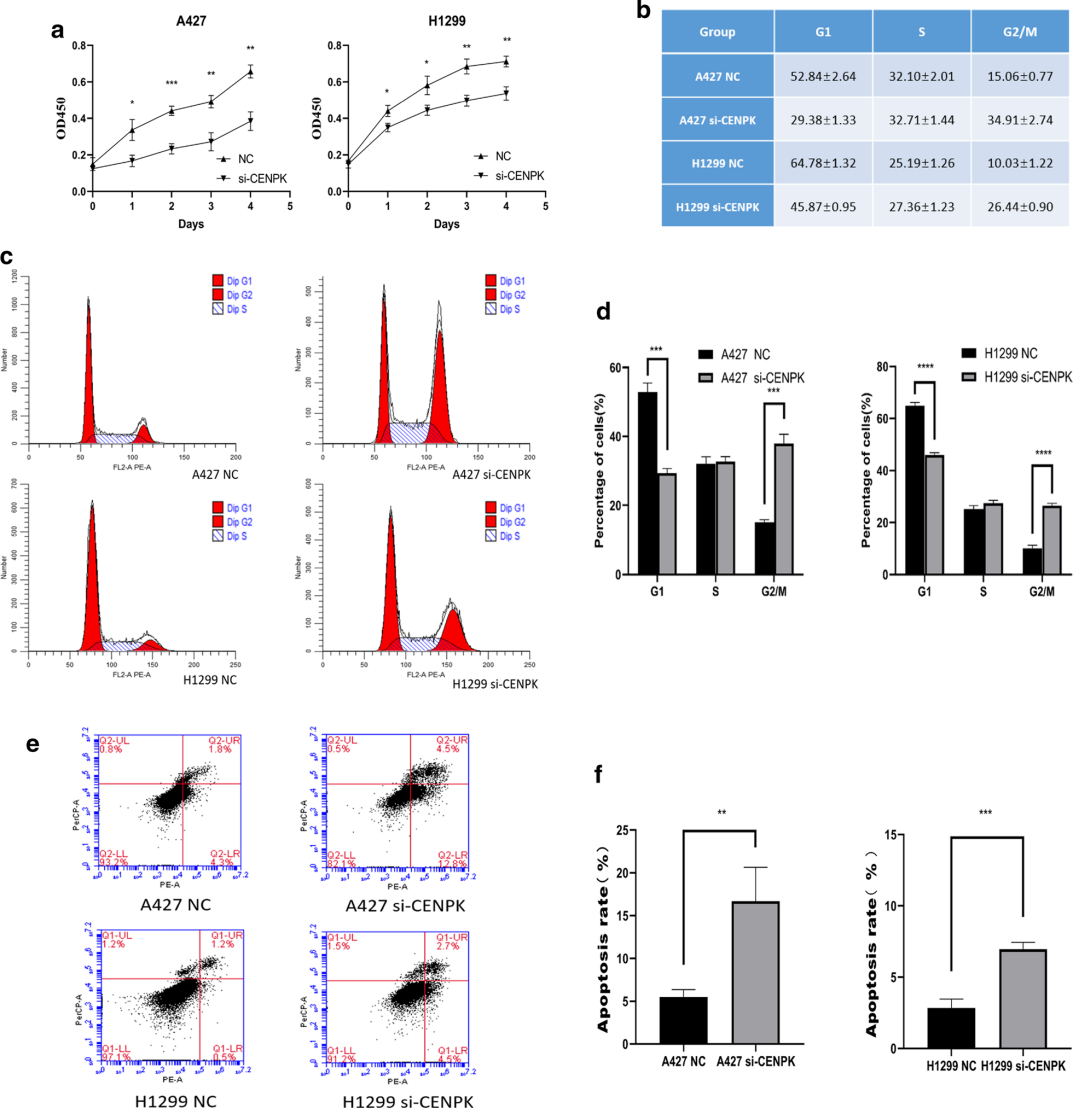
Effect of CENPK on cell proliferation. **a** Results of CCK8 assay showing a decline in the proliferation ability of A427 and H1299 cells after CENPK-siRNA transfection. **b** Proportion of cells in each phase are expressed as mean ± standard deviation. **c**, **d** Cell cycle and statistical analysis showing significant G2 arrest after CENPK knockdown. **e**, **f** The trend and statistical analysis showing significant increase in apoptosis ability after si-CENPK transfection

The results of CCK8 assay showed significant decline in the proliferation of A427 and H1299 cells after CENPK knockdown Fig. 9a. In the cell cycle experiments, the specific cell cycle phase ratio is shown in Fig. 9b, the trend of CENPK on the cell cycle is presented in Fig. 9c, and the statistical analysis is shown in Fig. 9d. The apoptosis ability of the si-CENPK transfection group was substantially increased Fig. 9e, the statistical analysis is shown in Fig. 9f. The findings illustrated that CENPK knockdown significantly inhibits cell proliferation.

### Knockdown of CENPK reduced cell invasion and migration

**Fig. 10:**
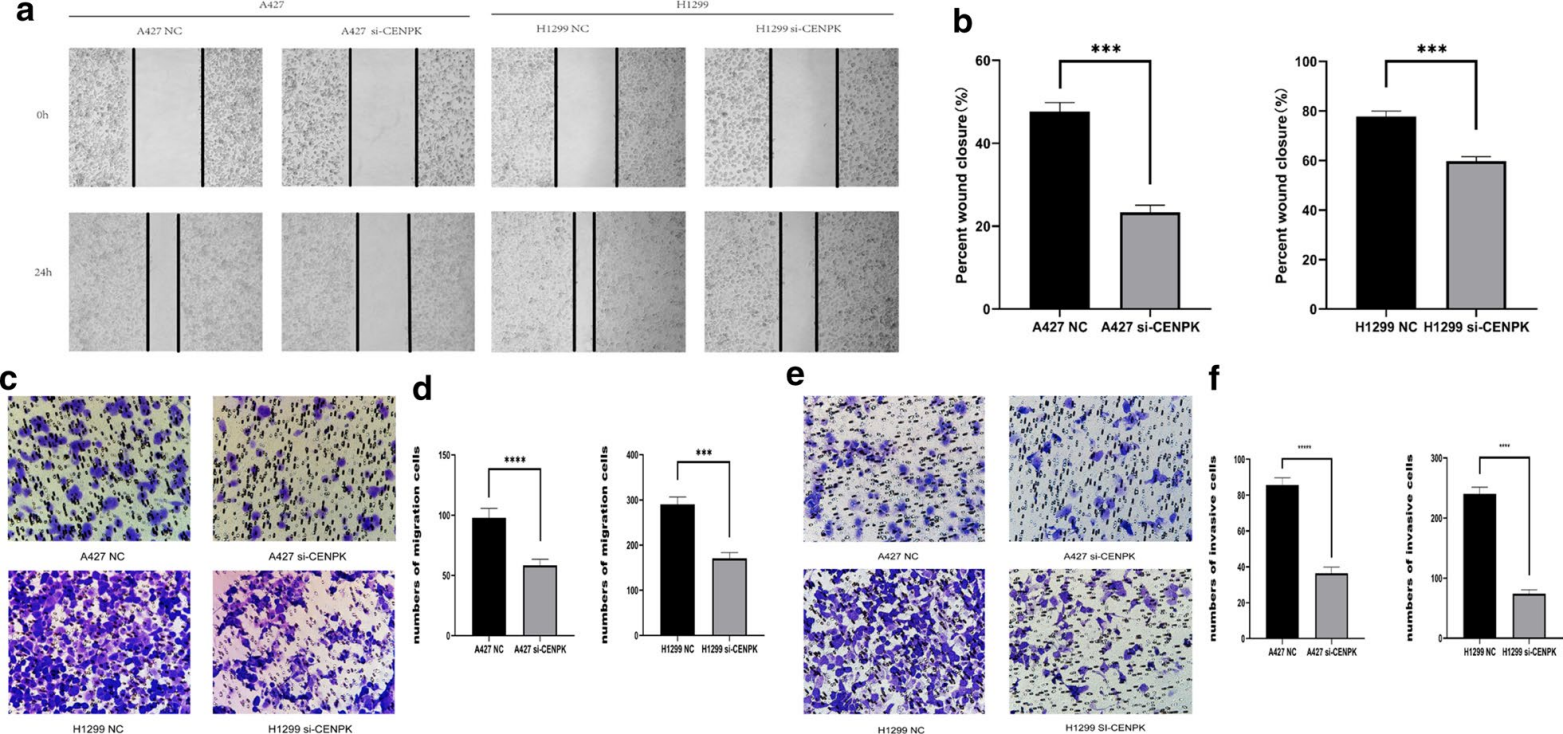
Effect of CENPK on cell invasion and migration. **a**, **b** Results of wound healing assay showing that si-CENPK inhibited the migration ability of A427 and H1299 cells. **c**, **d** The numbers of cells passing through the transwell membrane showing significant decrease in the migration and invasion ability after CENPK-siRNA transfection. **e**, **f** Results of transwell assay evaluating showing significant decrease in the invasion ability of cells after CENPK-siRNA transfection

Wound healing assay was performed to assess the effects of CENPK on cell migration; CENPK knockdown in A427 and H1299 cells significantly inhibited the migration ability Fig. 10a, b. This result was consistent with the results of transwell assay, the migration Fig. 10c, d and invasion Fig. 10e, f ability in the si-CENPK transgenic group was significantly suppressed.

## Discussion

Lung cancer is the most common cause of morbidity and mortality among all malignant tumors in China. In the past 20 years, LAC has been the most common subtype of NSCLC [23]. Till date, there are no standard prognostic biomarkers for LAC. Thus, biomarkers for early diagnosis and accurate prognostic assessment in LAC are essential to improve disease outcomes. This research aimed to combine bioinformatics analysis and basic experiments to identify gene with potential prognostic relevance. To the best of our knowledge, this is the first study to identify CENPK as a novel tumor marker of LAC.

CENP-K is a subunit of a constitutively centromere-associated complex, which is associated with the kinetochore assembly pathways [24]. The kinetochore, composed of centromere DNA and related proteins, plays an essential role in cell division and chromosomal segregation [25]. Several pieces of evidence have suggested the relation of CENPK with carcinogenesis and cancer progression. Previous studies have described the prognostic significance of CENPK for certain cancers. Up-regulation of CENPK was shown to be potentially regulated by epigenetic events and contribute to the development of hepatocarcinoma [14, 15]. Recently, several studies have investigated the biological activities of CENPK in tumor progression and metastasis. For example, CENPK was shown to be upregulated in ovarian cancer and its upregulation was related to poor prognosis [16]. Overexpression of CENPK was associated with poor outcomes in patients with triple-negative breast cancer [17].

In this study, we found significant upregulation of CENPK in LAC. Besides, CENPK expression in LAC was related to cancer stage, T stage and distant metastasis. In addition, high mRNA expressions of CENPK were associated with poor OS. Moreover, on multivariate Cox analysis, high CENPK expression was found to be an independent risk factor for OS of patients with LAC.

To the best of our knowledge, the role of CENPK in lung cancer has not been reported. To further verify the cellular mechanism of CENPK, we conducted functional experiments in A427 and H1299 cells to investigate the biological behavior of CENPK. In vitro, CENPK knockdown resulted in significant reduction in cell proliferation; flow cytometric analysis revealed cell arrest in G2/M phase, while the apoptotic capacity was significantly increased in the transfection group. In the wound healing assay, reversing the CENPK gene was found to significantly inhibit the migration capacity; results of transwell assay confirmed this result. The above results show that the CENPK knockdown inhibited the proliferation, migration, and invasive capacity of lung cancer cells.

We performed a pathway enrichment analysis of CENPK-related genes and identified some pathways; the results showed an association of CENPK over-expression with tumor progression and poor outcomes. We knocked down CENPK and found a significant association with cyclinB1, a key protein in the cell cycle; activation of cyclinB1 triggers the transition from G2 to mitosis. Moreover, on cell cycle analysis by flow cytometry, cells treated with si-CENPK were found to exhibited a G2/M phase arrest. CyclinB1 overexpression has been found in a variety of human tumors; its upregulation is closely related to poor outcomes in the context of breast cancer, cervical cancer, gastric cancer, colorectal cancer, head and neck squamous cell carcinoma, and NSCLC [26–33].

## Conclusions

In conclusion, our findings suggest that CENPK may serve as a potential diagnosis and prognostic biomarker in patients with LAC. This is the first study to investigate the expression of CENPK and changes in biological behavior in combination with bioinformatics analysis and experiments. Our study also has certain limitations. The research was validated in TCGA and GEO databases, followed by in vitro experiments; however, we did not perform relevant in vivo experiments. Additionally, our research has identified some potential pathways; further studies are required to investigate the underlying mechanisms.

## Supplementary Information

**Additional file 1: Table S1.** The statistics of patient information in the TCGA database. **Table S3.** The statistics of patient information in the GEO database. **Table S6.** The statistics of patient information in the immunohistochemistry slides.

**Additional file 2: Table S2.** The details of patient information in the TCGA database.

**Additional file 3: Table S4.** The details of patient information in the GEO database.

**Additional file 4: Table S5.** The details of patient information in the immunohistochemistry slides.

**Additional file 5: Figure S1.** Protein expression changes in cell cycle related proteins. Western blot analysis of cell cycle related proteins in NC and si-CENPK in A427 and H1299 cell lines. cyclinA2 (proteintech, catalog no. 18202-1-AP); cyclinD1 (proteintech, catalog no. 60186-1-Ig); cyclinE1 (proteintech, catalog no. 11554-1-AP); CDK1 (proteintech, catalog no. 10762-1-AP); CDK4 (proteintech, catalog no. 11026-1-AP).

## Data Availability

The datasets generated and/or analyzed during the current study are available in the [TCGA] repository [https://tcgadata.nci.nih.gov/tcga/] [34] and [GEO] repository [http://www.ncbi.nlm.nih.gov/geo] [35]. The datasets used and/or analyzed during the current study are available from the corresponding author on reasonable request.

## Abbreviations

LAC: Lung adenocarcinoma
CENPK: Centromere protein K
TCGA: The Cancer Genome Atlas
GEO: Gene Expression Omnibus
NSCLC: Non-small cell lung cancer
SCLC: Small cell lung cancer
GO: Gene ontology
KEGG: Kyoto encyclopedia of genes and genomes
HBE: Human bronchial epithelial
HR: Hazard ratio
H-score: Histochemistry score
NC: Negative control
