# Supplementary material for: Bioinformatics analysis combined with experiments predicts CENPK as a potential prognostic factor for lung adenocarcinoma
Source: Cancer Cell Int. 2021 Jan 21;21:65. doi: 10.1186/s12935-021-01760-y (PMC7818917; doi:10.1186/s12935-021-01760-y)
Supplement: Supplementary file 1 — Additional file 1: Table S1. The statistics of patient information in the TCGA database. Table S3. The statistics of patient information in the GEO database. Table S6. The statistics of patient information in the immunohistochemistry slides. [file 12935_2021_1760_MOESM1_ESM.docx]

**Supplementary Table 1. The statistics of patient information in the TCGA database.**

| Clinical characteristics | Total | % |
| --- | --- | --- |
| Age at diagnosis, years |  |  |
| <65 | 193 | 48.4 |
| >65 | 206 | 51.6 |
| Sex |  |  |
| Male | 181 | 45.4 |
| Female | 218 | 54.6 |
| Stage |  |  |
| I | 212 | 53.1 |
| II | 99 | 24.8 |
| III | 67 | 16.8 |
| IV | 21 | 5.3 |
| T stage |  |  |
| T1 | 139 | 34.8 |
| T2 | 208 | 52.1 |
| T3 | 36 | 9.1 |
| T4 | 16 | 4.0 |
| N stage |  |  |
| N0 | 256 | 64.2 |
| N1 | 81 | 20.3 |
| N2 | 60 | 15.0 |
| N3 | 2 | 0.5 |
| M stage |  |  |
| M0 | 272 | 68.2 |
| M1 | 18 | 4.5 |
| Mx | 109 | 27.3 |

**Supplementary Table 2. The statistics of patient information in the GEO database.**

Dataset: GSE72094 Platforms: GPL15048 Tumor: 398

| Clinical characteristics | Total | % |
| --- | --- | --- |
| Age at diagnosis, years |  |  |
| <65 | 118 | 29.6 |
| >65 | 280 | 70.3 |
| Sex |  |  |
| Male | 176 | 44.2 |
| Female | 222 | 55.8 |
| Stage |  |  |
| I | 239 | 60.1 |
| II | 62 | 15.6 |
| III | 61 | 15.3 |
| IV | 16 | 4.0 |
| Smoking statue |  |  |
| Never | 31 | 7.8 |
| Ever | 301 | 75.6 |

**Supplementary Table 6. The statistics of patient information in the immunohistochemistry slides.**

|  |  | | | CENPK expression | | total |
| --- | --- | --- | --- | --- | --- | --- |
|  |  | | | low | High |  |
| Age | | | <60 | 5 | 11 | 16 |
|  |  |  | >60 | 6 | 9 | 15 |
|  |  |  | Total | 11 | 20 | 31 |
| Sex | | | Male | 3 | 11 | 14 |
|  |  |  | Female | 8 | 9 | 17 |
|  |  |  | Total | 11 | 20 | 31 |
| Site | | | Right lung | 7 | 10 | 17 |
|  |  |  | Left lung | 3 | 11 | 14 |
|  |  |  | Total | 10 | 21 | 31 |
| Differentiation | | | Low | 1 | 5 | 6 |
|  |  |  | Middle | 4 | 8 | 12 |
|  |  |  | High | 6 | 7 | 13 |
|  |  |  | Total | 11 | 20 | 31 |
| Maximum diameter(cm) | | <3 | | 3 | 5 | 8 |
|  |  | >3 | | 7 | 16 | 23 |
|  |  | Total | | 10 | 21 | 31 |
| N stage | | N0 | | 8 | 9 | 17 |
|  |  | N1-2 | | 3 | 11 | 14 |
|  |  | Total | | 11 | 20 | 31 |
| T stage | | T1 | | 1 | 1 | 2 |
|  |  | T2 | | 7 | 16 | 23 |
|  |  | T3 | | 2 | 4 | 6 |
|  |  | Total | | 10 | 21 | 31 |
