# Supplementary figures and images for: Bioinformatics analysis combined with experiments predicts CENPK as a potential prognostic factor for lung adenocarcinoma
Source: Cancer Cell Int. 2021 Jan 21;21:65. doi: 10.1186/s12935-021-01760-y (PMC7818917; doi:10.1186/s12935-021-01760-y)

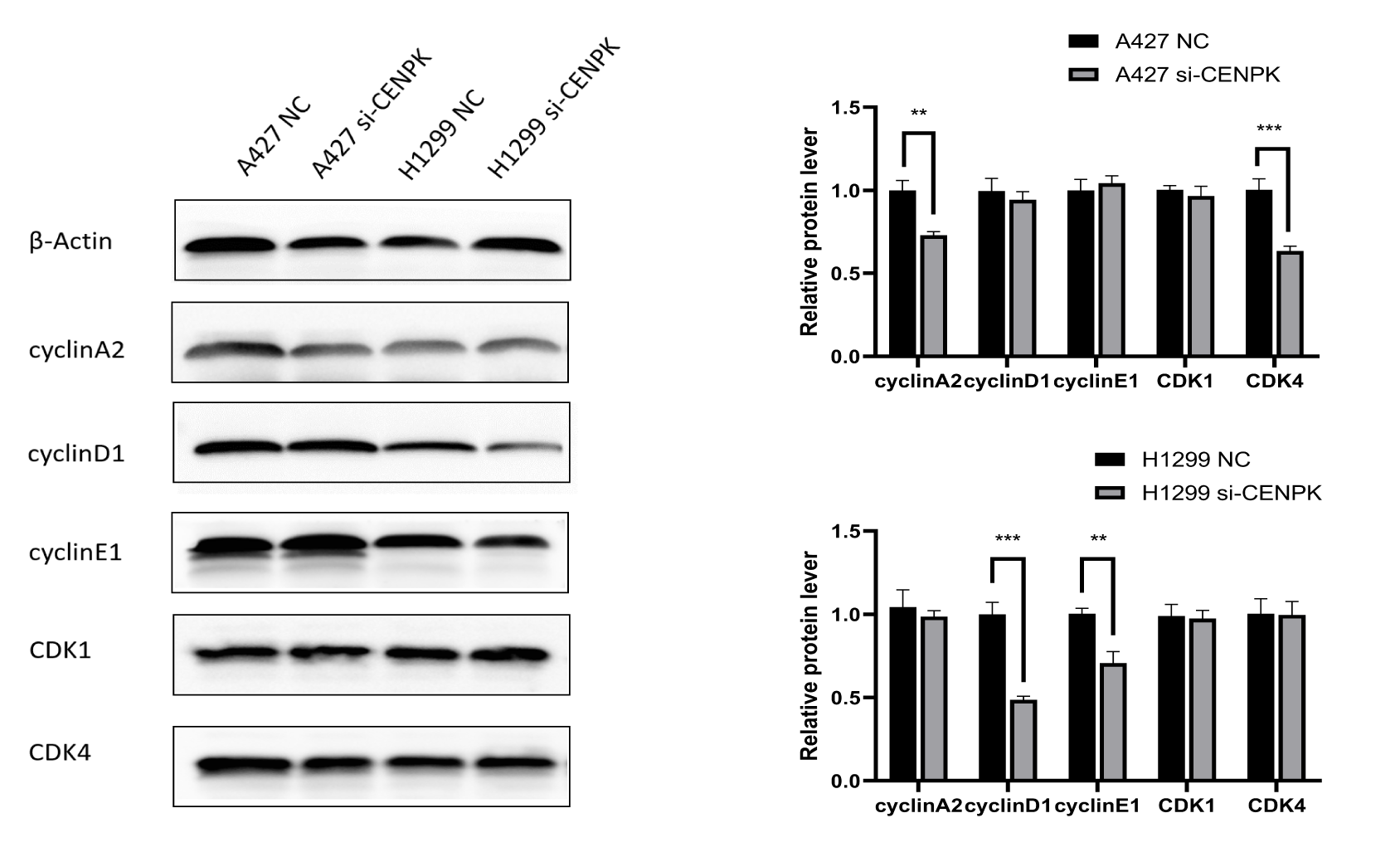

Supplement: Supplementary file 5 — Additional file 5: Figure S1. Protein expression changes in cell cycle related proteins. Western blot analysis of cell cycle related proteins in NC and si-CENPK in A427 and H1299 cell lines. cyclinA2 (proteintech, catalog no. 18202-1-AP); cyclinD1 (proteintech, catalog no. 60186-1-Ig); cyclinE1 (proteintech, catalog no. 11554-1-AP); CDK1 (proteintech, catalog no. 10762-1-AP); CDK4 (proteintech, catalog no. 11026-1-AP). [file 12935_2021_1760_MOESM5_ESM.tif]
